# The Compatibility of Children with Obesity to Self-Report Aspects of Physical Activity Domains

**DOI:** 10.3390/children9111664

**Published:** 2022-10-31

**Authors:** Liraz E. Anavi, Einat Kodesh, Gur Mainzer

**Affiliations:** 1 The Azrieli Faculty of Medicine, Bar-Ilan University, Safed 1311502, Israel; 2 Center for Exercise Medicine, Baruch Padeh Medical Center, Poriya 15208, Israel; 3 Physical Therapy Department, University of Haifa, Haifa 3498838, Israel; 4 Pediatric Heart Institute, Edmond and Lily Safra Children’s Hospital, Sheba Medical Center, Tel Hashomer 52621, Israel

**Keywords:** children self-report, obese, cardiopulmonary exercise testing

## Abstract

Questions about the different aspects of physical activity (PA) are commonly asked in the clinical setting, yet their compatibility for use with children, particularly children with obesity (OB) is uncertain. Our aim was to investigate different PA-related questions when compared to an objective maximal cardiopulmonary exercise test (CPET) or validated questionnaires. For this study, 33 normal-weight (NW) (5 to less than 85% BMI percentile) and 35 OB (≥95% BMI percentile) children responded to three self-report PA questions evaluating PA domains (exercise capacity, limitations, and the maintenance of an active lifestyle); they also completed a maximal CPET and two validated questionnaires: the New York Heart Association (NYHA) questionnaire and the international physical activity questionnaire (IPAQ). The results regarding the NW children were highly compatible with their self-reports about exercise capacity (85%), whereas the compatibility was low (40%) in the OB group (*p* < 0.001). Both OB and NW groups had moderate compatibility between the self-report and objective findings regarding their exercise limitations and lifestyle with no significant differences between the groups. These findings suggest that it is inadvisable to rely on a single-item question by which to assess PA in OB children, and no definite conclusions regarding PA status should be drawn. NW children are more compatible with self-reporting their overall exercise capacity, with more limited compatibilities observed when self-reporting their limitations or lifestyle.

## 1. Introduction

The prevalence of worldwide obesity has tripled since 1975. Internationally, 38 million children under the age of 5 and over 340 million children and adolescents aged 5-19 were found to be obese in 2016 [1]. Obese (OB) children are at increased risk of adult obesity and are more likely to experience physical and mental health complications, such as cardiovascular illnesses, hyperlipidemia, glucose intolerance, and low self-esteem, in comparison to their normal-weight (NW) peers [2,3,4]. There are multiple risk factors contributing to the development of childhood obesity and its associated pathologies; however, one modifiable major risk factor is the lack of physical activity [2,4]. Thus, in order to screen and determine the correct interventions to improve physiological and psychological outcomes, an accurate and reliable assessment of physical activity (PA) is required in OB children [2,3].

Many approaches are used to evaluate the different aspects of PA, including self-report questionnaires, direct observations, heart rate monitors, motion sensors, and exercise tests, such as the cardio-pulmonary exercise test (CPET) [2,3]. In spite of the fact that objective measurements are more accurate in the assessment of physical activity, the high cost and demanding methodology of these methods render them unsuitable for routine clinical use. Consequently, their relatively low cost, ease of administration, and their ability to cover details of physical activity over a period of time have led physicians to rely on single-item questions to evaluate PA domains in clinical settings [3,5,6].

Physicians tend to consider two factors when implementing PA self-reports: (1) the limited time that they have for each patient encounter, as presented by Overhage and McCallie [7], and (2) the compatibility of patients when self-reporting different aspects of PA, which is known to be affected by age, gender, and BMI [8]. 

Few studies have investigated the compatibility of self-evaluating physical activity via a single question [6]. 

Peterson et al. [9] examined the correlation and association between self-rated physical fitness via a single question in adults, “How do you rate your own physical fitness?”, and an objective measure of cardiorespiratory fitness, measured by a maximal exercise test; they concluded that in adults who were 18–85 years of age, this question can be utilized as a tool to identify and monitor changes in cardiorespiratory fitness. 

In general, the self-reporting of physical activity had low compatibility compared to objective measurements in children with obesity [10,11]. Elliot et al. [10] reported that OB children completing a four-day self-report diary overestimated their physical activity, in correlation with doubly labeled water measures. Jackicic et al. [11] concluded that there was a weak to moderate correlation between a variety of PA questionnaires to accelerometer measurements in OB children and adolescents. There is a lack of evidence regarding the compatibility of a single-item question in children. Due to the limited clinical time and lack of certainty regarding the compatibility of PA self-reports, especially for single-item PA questions, it is essential to examine their compatibility with both healthy and OB children. 

Our aim was to examine the compatibility of three single questions related to three PA domains (exercise capacity, limitations, and the maintenance of an active lifestyle), compared to objective or validated PA assessment methods: the percentage of maximal predicted oxygen consumption (%VO_2peak pred_) collected according to the cardiopulmonary exercise test (CPET) parameters, the New York Heart Association (NYHA) questionnaire, and the international physical activity questionnaire (IPAQ), in both OB and normal-weight (NW) children. We hypothesized that the OB children would have lower compatibility than their NW counterparts.

## 2. Materials and Methods

### 2.1. Design

In this cross-sectional study, we assessed the compatibility of OB and NW children regarding self-reporting exercise domains related to physical activity by asking three single-PA domain questions. These three questions are commonly asked during routine clinical evaluation and include questions about exercise capacity (“Do you have difficulties climbing up a flight of stairs? Yes, or no”), exercise limitations (“Do you have any limitations while performing physical activities (dizziness, difficulty breathing, fatigue, muscle pain)? Yes or no”), and the maintenance of an active lifestyle (“Do you maintain an active lifestyle (walking to school, playing with friends, climbing up the stairs)? Yes or no”). Data were collected as part of a single visit to the Center of Exercise Medicine in Baruch Padeh for cardiopulmonary assessment. The study was approved by the Baruch Padeh Hospital Helsinki committee (protocol number 0033-17-POR), and written consent was obtained from the pediatric patients and their legal guardians (ClinicalTrials.gov Identifier: NCT05205642).

### 2.2. Participants

Sixty-eight children aged 6–18 underwent a physical activity assessment at the Center of Exercise Medicine of Baruch Padeh Hospital between 2019 and 2021. The inclusion criteria were individuals aged 6–18, with no known diseases or pathologies. BMI percentiles were calculated according to the “Center for Disease Control and Prevention” BMI-for-age growth charts [12,13]. The 35 children (10 girls) with a BMI in the 95th percentile or above were included in the obese (OB) group, while 33 children (20 girls) with a BMI between the 5th and less than 85th percentile were defined as normal-weight (NW) and served as controls. 

### 2.3. Data Collection

On the day of the clinical examination, demographic (gender and age (and anthropometric (height, weight, abdominal waist circumference, body mass index (BMI), and percentile) data were collected, a cardiopulmonary exercise test (CPET) was performed, and two validated PA-self reports and three routine PA questions were administered.

#### 2.3.1. Exercise Testing with CPET

The CPET [14] was performed with a metabolic cart (Quark, Cosmed, Italy), according to the American Thoracic Society guidelines [15], on a treadmill using the Bruce ramp protocol. During the Bruce ramp protocol, the treadmill speed or slope or both increased every minute until the participant reached exhaustion. For the purposes of this study, we utilized the peak oxygen consumption (VO_2_), expressed as a percentage of the maximal predicted (%VO_2peak pred_), to measure the participant’s exercise capacity. Normal exercise capacity was defined as %VO_2peak pred_ ≥ 80%. All participants were encouraged to exercise to their maximal ability.

#### 2.3.2. New York Heart Association (NYHA) Classification

The NYHA classification [16] stratifies the participants into one of four classes, according to their level of dyspnea during physical activities. The four classes range from “no shortness of breath” to “shortness of breath during rest”. In the current study, we used two categories: participants with no shortness of breath (NYHA I) and those with any level of shortness of breath (NYHA II–IV).

#### 2.3.3. International Physical Activity Questionnaire (IPAQ)

The IPAQ [17] evaluates 4 levels of activity that have been performed over the seven days before the questionnaire is administered: vigorous intensity, moderate intensity, walking, and sitting. The total number of days and minutes of physical activity during the week is then calculated and is then converted into metabolic equivalents per week (METs wk^−1^) for each participant. The results of the IPAQ are usually divided into three categories, according to the calculated METs wk^−1^ (high, moderate, and low). Scoring a moderate level of physical activity on the IPAQ is defined as achieving a minimum total physical activity of at least 600 MET minutes per week [17]. Thus, in the current study, we stratified the participants into two categories: active (≥600 METs wk^−1^) and inactive (<600 METs wk^−1^).

#### 2.3.4. Single-Item Questions

The participants were asked three questions, similar to those used in previously published studies and commonly asked in the clinic to evaluate exercise capacity, exercise limitations, and the maintenance of an active lifestyle:

(Q1) Exercise capacity: “Do you have difficulties climbing up a flight of stairs? Yes, or no”—inspired by the stair climbing test [18];
(Q2) Exercise limitations: “Do you have any limitations while performing physical activities (dizziness, difficulty breathing, fatigue, muscle pain)? Yes or no”—based on items from the physical activity readiness questionnaire for everyone (PAR-Q+) [19];
(Q3) Maintenance of an active lifestyle “Do you maintain an active lifestyle (walking to school, playing with friends, climbing up the stairs)? Yes or no”—based on the physical activity component of the speedy nutrition and physical activity assessment (SNAP) [20].

### 2.4. Compatibility Levels

The compatibility of exercise capacity (Q1), exercise limitations (Q2), and the maintenance of an active lifestyle (Q3) was defined as “acceptable” when the responses corresponded to the objective results of the CPET %VO_2peak pred_ and validated scores from the NYHA classification and IPAQ questionnaire. 

The compatibility of Q1 for assessing exercise capacity was defined as a response of “no” with a corresponding %VO_2peak pred_ ≥ 80%, or “yes” with a corresponding %VO_2peak pred_ < 80%. 

The compatibility of question Q2 for assessing exercise limitations was defined as a response of “no” with an NYHA score of I, or “yes” with a corresponding NYHA score of II–IV. 

The compatibility of Q3 for assessing the maintenance of an active lifestyle was defined as a response of “no” with a corresponding IPAQ category of inactive (<600 METs wk^−1^), or “yes” with a corresponding IPAQ category of active (≥600 METs wk^−1^), respectively. 

### 2.5. Statistical Analysis

Descriptive statistical analyses were conducted for each group using the SPSS version 27 software. Variables are presented as mean and standard deviation or as a proportion for the categorical variables. Student’s *t*-test was conducted to determine the significant differences between the two groups for anthropometric, demographic, exercise, and physical activity parameters. The size effect was calculated using Cohen’s d method. 

The NYHA, IPAQ, and %VO_2peak pred_ were converted into categorical variables, as described above, and a chi-square test was conducted to examine any significant differences between the OB and NW groups. Compatibility was calculated as the proportion of the number of members in a group meeting a criterion out of the total number of group members. Cohen’s h [21] was calculated for measuring the effect size for the chi-square test of the total compatibility of each question. The value *p* < 0.05 was considered to be statistically significant.

## 3. Results

Sixty-eight pediatric patients met the inclusion criteria for the study. The mean values of the demographic, anthropometric, and exercise parameters of the two groups are presented in Table 1**.** The NW group included more females (20 (60.6%) against the OB group of 10 (28.6%); <0.001) and the OB group had a significantly lower %VO_2peak pred_ than the NW group.

### 3.1. The Compatibility of the NW and OB Participants for Self-Reporting Three Exercise Domains

The compatibility of the NW and OB participants was assessed by comparing the responses to the three questions to the validated measures, as shown in Table 2, Table 3 and Table 4.

#### 3.1.1. Exercise Capacity Assessment 

The exercise capacity self-report, compared to the answers to Q1 with %VO_2peak pred_, as presented in Table 2, demonstrates a significant difference in total compatibility between the groups (*p* < 0.001). The NW group showed high compatibility (84.8%), predominantly in those who responded “no” (87.1%), while the OB group showed no compatibility with self-reported exercise capacity. A small minority of OB participants responded “yes” to Q1 and had perfect compatibility (100%) with %VO_2peak pred_. The total compatibility size effect (Cohen’s h) was 0.97, indicating a large size effect. 

#### 3.1.2. Exercise Limitation Assessment

The exercise limitation was evaluated for Question Q2, corresponding to the NYHA (Table 3). Evaluating the compatibility of Q2 with the NYHA demonstrated moderate compatibility in both groups, with no significant differences. Specifically, the “No” responders (30 NW and 21 OB) displayed high compatibility (80.0% and 85.7%, respectively). 

The total compatibility size effect (Cohen’s h) was 0.15, indicating a small size effect.

#### 3.1.3. Active Lifestyle Assessment

The correspondence with the maintenance of an active lifestyle and self-reporting, examined by Q3 of the IPAQ questionnaire, is presented in Table 4. The NW and OB participants showed moderate compatibility (NW 63.6% and OB 74.3%), with no statistically significant difference between the groups. The total compatibility size effect (Cohen’s h) was 0.23, indicating a small size effect.

## 4. Discussion

Our study examined the compatibility of the answers to three single-item questions regarding an objective exercise test and to the validated questionnaires completed by OB and NW children and found that there was limited compatibility in the answers of children to questions about exercise capacity, exercise limitations, and an active lifestyle. Lower compatibility was found in the OB group compared to the NW group. To the best of our knowledge, this is the first study on the compatibility of OB children regarding their response to a single question about their exercise capacity, versus VO_2peak pred_. The compatibility with self-reporting exercise capacity, as evaluated by question Q1, “Do you have difficulties climbing up a flight of stairs?”, was inferior in OB participants, with only 40% compatibility, compared to ~85% of the NW. This highlights the finding that the answers provided by OB children about exercise capacity are unreliable. Interestingly, the OB group had 100% compatibility when reporting difficulties. Aadahl et al. [22] found that the response of 102 participants aged 35–65 years, rating their fitness using a single-item question as excellent, very good, good, fair, or poor, reflected their VO_2max_ when measured on a CPET with a bike ergometer. De Moraes et al. [23] tested the agreement of self-reported physical fitness by examining the cardiorespiratory fitness component of the international fitness scale in comparison to a physical test in NW children and reported an 86.2% agreement in children and 81.82% agreement in adolescents. Conversely, Barber investigated the compatibility of the subjective estimation of exercise capacity in 211 NW children and found only 30% compatibility with the VO_2max_ results [24]. Similar findings came from a study comparing the accuracy of reporting by children with congenital heart diseases of various complexity when subjectively rating their exercise capacity, using one of five possibilities (very good, good, moderate, bad, and very bad) to their VO_2peak pred_, and concluded that the answers of children with chronic disabilities were unreliable and they tended to overestimate their exercise capacity [25]. This overestimation of exercise capacity may occur in other individuals with chronic limitations, e.g., OB individuals, because prolonged adaptation to their disabilities has made them unaware of their true exercise capacity [25,26]. 

The straightforward question about limitations during physical activity, when compared to the NYHA score, had a compatibility of 72.2% and 65.7% in the NW and OB groups, respectively. Interestingly, those declaring “no limitations” yielded a reliable answer in both groups (80% and 85% in the NW and OB groups, respectively), whereas the self-reporting of limitations demonstrated poor compatibility in both groups (0 and 35.7% in the NW and OB groups, respectively). Although the NYHA classification was created to evaluate exercise ability in adults with chronic heart failure, it has become a widely used and validated method to classify individuals, especially adults, into one of four classes, based on the subjective estimation of physical limitations, and has been used in children with congenital heart diseases [26,27]. The NYHA functional classification places patients into categories, based on palpitation, dyspnea, and the perception of fatigue, and discomfort. This may suggest that the use of the NYHA classification in children is not sensitive enough to evaluate their limitations, and may be expressed better using other domains, such as muscle soreness and perceived exertion, and needs further investigation [26,28,29,30]. 

We found moderate compatibility (60–70%) in both groups when evaluating the maintenance of an active lifestyle. in comparison with IPAQ, showing no differences between the groups. Similar findings were reported by Galfo [31], where a low level of agreement (r = 0.32) was found between a self-administered physical activity questionnaire and accelerometer data in adolescents. Milton [32] reported an overall agreement of 58% between a single-item question about the number of days including ≥30 min of moderate to vigorous PA and the accelerometer data, regarding classifying participants as active or inactive. Using accelerometer data as a comparison, Ball [20], gave adults the physical activity vital sign (PAVS) questionnaire, designed to assess the past and a typical week of moderate to vigorous physical activity, and found that the PAVS questionnaire showed only moderate agreement, as shown by a Kappa degree of agreement of 0.46 with the accelerometers. The past year’s total physical activity questionnaire, which assesses the type of activity as well as the frequency, duration, and perceived intensity of the activity over the past year, was given to adults with obesity and Ferrari et al. [33] concluded that individuals with a high BMI had low accuracy in self-reported physical activities in comparison to accelerometer measurements.

### Limitations

Our study had a significant gender difference between the groups (60.6% females in the NW group and 28.6% females in the OB group) and these differences may have influenced our results. According to Sallis et al. [3], males are more reliable self-reporters than females. Furthermore, the unequal distribution between the “yes” and “no” answers, and the relatively small sample size limits our ability to make definite conclusions about those answering “yes” or “no”. Finally, as far as we know no validated questionnaire assessing exercise limitation exists, therefore we used the NYHA, which has been found to be less appropriate for children [26,28]. 

## 5. Conclusions

Our study highlights the limitations of using answers to a single question to evaluate specific PA domains in children, along with the lack of compatibility when posing such questions to children with obesity. The assessment of physical activity domains is important, especially in a clinical setting, where the information may affect the selection of a physical activity intervention program, medication dose, or invasive treatment. Our findings encourage physicians, parents, teachers, and those who are involved in decision-making regarding health behavior in this population to use objective tests and validated questionnaires, in order to learn about the physical activity domains of children in general and specifically in those who are medically compromised.

## Figures and Tables

**Table 1:** Demographic, anthropometric, exercise, and physical activity parameters.

	NW (*n* = 33)	OB (*n* = 35)	*p*-Value	Cohen’s d Effect Size
Age (years)	12.6 ± 4	11.7 ± 2.67	0.62	0.24
BMI (%)	60 ± 22	99 ± 1	<0.001	−2.78
Abd Cir. (cm)	66 ± 8.4	98.7 ± 20.5	<0.001	−2.07
VO_2peakpred_ (%)	92 ± 14.5	66 ± 16	<0.001	1.80
IPAQ (METs wk^−1^)	2175 ± 2640	1179 ± 1677	0.06	0.49

Data are presented as mean ± SD. Abd Cir-abdominal circumference; BMI-body mass index; IPAQ-international physical activity questionnaire; METs wk^−1^-metabolic equivalents per week; NW-normal weight; OB-obese; VO_2peak pred_.-peak oxygen consumption/predicted peak oxygen consumption. A *p*-value < 0.05 represents statistically significant differences between the group. Cohen’s d effect size: *d* = 0.2, is considered a small effect; *d* = 0.5 is considered a moderate effect; *d* > 0.8 is considered a strong effect.

**Table 2:** Exercise capacity assessment, showing the compatibility of Question 1 to %VO_2peak pred_.

Question 1		NW		OB		
	Response	*n*	*Comp. *n* (%)	*n*	*Comp. *n* (%)	*p*-Value
Do you have difficulties climbing up a flight of stairs?	No	31	27 (87.1)	28	7 (25.0)	<0.001
	Yes	2	1 (50.0)	7	7 (100.0)	0.06
	Total	33	28 (84.8)	35	14 (40.0)	<0.001

*n*-number of participants; Comp.-compatible; NW-normal weight; OB-obese; %VO_2peakpred_-the percentage of predicted maximal oxygen consumption. *Comp. (%) corresponds to the number of patients responding “No” with a %VO_2peak pred_ ≥ 80%, or to the number of patients. responding “Yes” with a %VO_2peak pred_ < 80%. *p*-value < 0.05 represents statistically significant differences between the groups.

**Table 3:** Exercise limitation assessment, showing the compatibility of Question 2 with the NYHA.

		NW		OB		
Question 2	Response	*n*	*Comp. *n* (%)	*n*	*Comp. *n* (%)	*p*-Value
Do you have limitations while performing physical activities?	No	30	24 (80.0)	21	18 (85.7)	0.60
	Yes	3	0 (0)	14	5 (35.7)	0.23
	Total	33	24 (72.7)	35	23 (65.7)	0.61

*n*-number of participants; Comp.-compatible; NW-normal weight; NYHA-New York Heart Association; OB-obese. *Comp. (%) corresponds to the number of participants responding “No”, with an NYHA score of I, or to the number of participants responding “Yes”, with an NYHA score of II–IV. A *p*-value < 0.05 represents statistically significant differences between the groups.

**Table 4:** Active lifestyle assessment, showing the Compatibility of Question 3 to the IPAQ.

		NW		OB		
Question 3	Response	*n*	*Comp. (%)	*n*	*Comp. (%)	*p*-Value
Do you maintain an active lifestyle?	No	3	1 (33.3)	5	4 (80.0)	0.22
	Yes	30	20 (66.7)	30	22 (73.3)	0.60
	Total	33	21 (63.6)	35	26 (74.3)	0.34

*n*-number of participants; Comp.-compatible; IPAQ-international physical activity questionnaire; NW-normal weight; OB-obese. *Comp. (%) corresponds to the number of participants responding “No” that were inactive (according to the IPAQ), or to the number of participants responding “Yes” that were active (according to the IPAQ). *p*-value < 0.05 represents statistically significant differences between the groups.

## Data Availability

The data presented in this study are available on request from the corresponding author.
